# A precision overview of genomic resistance screening in Ecuadorian isolates of *Mycobacterium tuberculosis* using web-based bioinformatics tools

**DOI:** 10.1371/journal.pone.0294670

**Published:** 2023-12-05

**Authors:** Gabriel Morey-León, Paulina M. Mejía-Ponce, Juan Carlos Granda Pardo, Karen Muñoz-Mawyin, Juan Carlos Fernández-Cadena, Evelyn García-Moreira, Derly Andrade-Molina, Cuauhtémoc Licona-Cassani, Luisa Berná

**Affiliations:** 1 Facultad de Ciencias de la Salud, Universidad Espíritu Santo, Samborondón, Ecuador; 2 Universidad de la República, Montevideo, Uruguay; 3 University of Guayaquil, Guayaquil, Ecuador; 4 Escuela de Ingeniería y Ciencias, Tecnológico de Monterrey, Monterrey, Nuevo León, México; 5 Centro de Referencia Nacional de Micobacterias, Instituto Nacional de Investigación en Salud Pública Dr Leopoldo Izquieta Perez, INSPI-LIP, Guayaquil, Ecuador; 6 Laboratorio de Ciencias Ómicas, Universidad Espíritu Santo, Sambor*o*ndón, Ecuador; 7 African Genome Center, University Mohammed VI Polytechnic (UM6P), Ben Guerir, Morocco; 8 Instituto Superior Tecnológico Argos, Guayaquil, Ecuador; 9 Laboratorio de Interacciones Hospedero-Patógeno, Unidad de Biología Molecular, Institut Pasteur de Montevideo, Montevideo, Uruguay; 10 Unidad de Genómica Evolutiva, Facultad de Ciencias, Universidad de la República, Montevideo, Uruguay; Universite Paris XI: Universite Paris-Saclay, FRANCE

## Abstract

**Introduction:**

Tuberculosis (TB) is among the deadliest diseases worldwide, and its impact is mainly due to the continuous emergence of resistant isolates during treatment due to the laborious process of resistance diagnosis, nonadherence to treatment and circulation of previously resistant isolates of *Mycobacterium tuberculosis*. In this study, we evaluated the performance and functionalities of web-based tools, including Mykrobe, TB-profiler, PhyResSE, KvarQ, and SAM-TB, for detecting resistance in 88 Ecuadorian isolates of *Mycobacterium tuberculosis* drug susceptibility tested previously. Statistical analysis was used to determine the correlation between genomic and phenotypic analysis. Our results showed that with the exception of KvarQ, all tools had the highest correlation with the conventional drug susceptibility test (DST) for global resistance detection (98% agreement and 0.941 Cohen’s kappa), while SAM-TB, PhyResSE, TB-profiler and Mykrobe had better correlations with DST for first-line drug analysis individually. We also identified that in our study, only 50% of mutations characterized by the web-based tools in the *rpoB*, *katG*, *embB*, *pncA*, *gyrA* and *rrs* regions were canonical and included in the World Health Organization (WHO) catalogue. Our findings suggest that SAM-TB, PhyResSE, TB-profiler and Mykrobe were efficient in determining canonical resistance-related mutations, but more analysis is needed to improve second-line detection. Improving surveillance programs using whole-genome sequencing tools for first-line drugs, MDR-TB and XDR-TB is essential to understand the molecular epidemiology of TB in Ecuador.

**Importance:**

Tuberculosis, an infectious disease caused by *Mycobacterium tuberculosis*, most commonly affects the lungs and is often spread through the air when infected people cough, sneeze, or spit. However, despite the existence of effective drug treatment, patient adherence, long duration of treatment, and late diagnosis have reduced the effectiveness of therapy and increased drug resistance. The increase in resistant cases, added to the impact of the COVID-19 pandemic, has highlighted the importance of implementing efficient and timely diagnostic methodologies worldwide. The significance of our research is in evaluating and identifying a more efficient and user-friendly web-based tool to characterize resistance in *Mycobacterium tuberculosis* by whole-genome sequencing, which will allow more routine application to improve TB strain surveillance programs locally.

## Introduction

Tuberculosis (TB), caused by *Mycobacterium tuberculosis* (Mtb), is one of the top 10 causes of death worldwide. In 2021, the World Health Organization (WHO) estimated that 10.6 million people were infected and 1.6 million people died [1]. Despite many innovations in tuberculosis diagnosis, drug resistance detection, drug therapy, prevention and control programs, several challenges, such as patient adherence, long duration of treatment, and late diagnosis, continue to limit the effectiveness of TB therapy [2]. The increase in resistant TB patients is not only due to exposure to multidrug-resistant (MDR) and extensively resistant strains (XDR) but also due to late or inadequate diagnosis (associated with slow growth of Mtb), ineffective treatment or poor adherence to treatment [2]. On the other hand, the accumulation of point mutations in coding regions for drug targets and/or drug-converting enzymes is a major mechanism for acquiring resistance in Mtb [3], which has further complicated the situation, making the timely detection of resistance-conferring mutations crucial for effective treatment and prevention of onwards transmission [4], for which the use of procedures that reduce the time of diagnosis is recommended.

The emergence of whole-genome sequencing (WGS) as a tool for detecting drug resistance in Mtb has revolutionized tuberculosis diagnosis and treatment [5]. Compared to traditional drug susceptibility testing, WGS provides results in a fraction of the time [6], making it a faster and more effective method for detecting resistance to all drugs simultaneously, identifying lineages, tracing transmission, and defining outbreaks [7–11]. With large datasets generated in tuberculosis research by WGS, the emergence of bioinformatics web-based tools such as TBProfiler [12], KvarQ [13], TGS-TB [14], Mykrobe Predictor TB [15], CASTB [16], PhyTB [17], ReSeqTB-UVP [18], GenTB [19], PhyResSE [20], SAM-TB [21] and others has significantly improved the efficiency of genotyping and drug resistance identification in *Mycobacterium tuberculosis*. These tools are popular due to their ease of use, ability to classify SNPs, feasibility of batch analysis, and user-friendly interfaces. However, they do have some technical limitations, such as the lack of standardized criteria for discerning noncanonical mutations, and selecting mutations with diagnostic criteria for resistance in new and repurposed antibiotics remains a challenge for utilizing WGS technology effectively [22]. Although web-based tools for TB lineage and resistance characterization have limitations, their combination with the WHO updated catalogue of resistance-associated mutations in TB [23] has enabled efficient implementation of WGS in surveillance programmes [24–26]. Despite these challenges, WGS technology offers reliable and efficient results that can guide TB diagnosis and treatment. The potential benefits of WGS in TB diagnosis and treatment make it a promising tool for improving public health policies and global TB control efforts.

The incidence of tuberculosis has been on the rise in many countries, including Ecuador, since 2015 [27]. The COVID-19 pandemic has added further complexity to this issue. The emergence of drug-resistant strains is a serious global threat and poses significant challenges to public health, particularly in low- and middle-income countries. This study aims to evaluate the effectiveness and capabilities of Mykrobe, TB-profiler, PhyResSE, KvarQ, and SAM-TB web-based tools for detecting drug resistance in *Mycobacterium tuberculosis* isolates with conventional DST. This evaluation is particularly important in the local context, where rapid diagnosis is critical for controlling multidrug-resistant tuberculosis (MDR-TB).

## Material and methods

A total of 88 clinical isolates were included in this study, obtained by convenience sampling from private laboratories and the National Reference of Mycobacteria at the National Institute of Public Health Research "Leopoldo Izquieta Pérez" (INSPI-LIP) in Guayaquil between 2019 and 2021. These isolates were identified as multidrug-resistant through DST agar [28] or the Bactec MGIT 960 System protocol [29] prior to their inclusion in the study. The resistance pattern of first-line and second-line anti-tuberculosis drugs was determined according to the proportional method by the Bactec MGIT 960 System protocol in 67 samples with critical concentrations of drugs as follows: rifampicin, 1.0 μg/mL; isoniazid, 0.1 μg/mL; ethambutol, 5.0 μg/mL; kanamycin, 2.5 μg/mL, capreomycin, 1.0 μg/mL, amikacin, 1.0 μg/mL, 1.0 μg/mL for levofloxacin and 1.0 μg/mL for moxifloxacin. However, in 21 samples, the resistance was determined by DST agar, and the critical concentrations of anti-TB drugs for the DST assays were as follows: rifampicin, 40.0 μg/mL; isoniazid, 0.2 μg/mL; ethambutol, 0.4 μg/mL; streptomycin, 4.0 μg/mL, and 200 μg/mL for pyrazinamide (pyrazinamidase assay). The resistance profile was defined based on the results from all isolates according to WHO recommendations.

### DNA extraction and sequencing

Genomic DNA was extracted from 88 isolates of *Mycobacterium tuberculosis* grown in Lowenstein-Jensen medium by two different protocols. In 21 isolates, the DNA was extracted by the CTAB method [30, 31], while for others, 67 were obtained by the PureLink DNA Mini Kit (Fisher Scientific, Pennsylvania, USA) according to the manufacturer’s instructions. Each extracted DNA sample was quantified by a Qubit 4.0 fluorometer (Invitrogen, Carlsbad, CA, USA.). DNA samples that fulfilled the quality standards in terms of integrity, purity and quantity were sequenced. Genomic DNA libraries were prepared for whole-genome sequencing using the Tagmentation-based library prep kit according to the manufacturer’s instructions (Illumina Inc., San Diego, CA, USA). Next, libraries with different indices were multiplexed and loaded on an Illumina MiniSeq platform with a High Output Reagent Kit, according to the manufacturer’s instructions (Illumina, San Diego, CA, USA). Sequencing was carried out using a 2 x 150 paired-end (PE) configuration.

### Bioinformatic analyses

Using the Galaxy platform (https://usegalaxy.org/), reads were classified by Kraken version 2 [32] to detect possible contamination or the presence of other mycobacteria. In addition, FastQC version 0.11.9 [33] and Trimmomatic version 0.38 [34] were used to control quality and trim the low-quality ends of the reads, respectively. In particular, a sliding window was used to trim sequences with an average quality value lower than 20. The high-quality reads were mapped to *M*. *tuberculosis* strain HR37v (NC_000962.3) using BWA-MEM [35]. To ensure high reliability, only isolates with sequences meeting strict criteria were included in the analysis. Specifically, any isolate with a sequencing depth of less than 20X or a reference coverage of less than 90% was excluded from subsequent analyses.

For phylogenetic analysis, clean reads were used as input in the MTBseq pipeline [36] to obtain i) sublineage classification, ii) transmission group identification and iii) SNP-based alignment for phylogenomic analysis. *Mycobacterium microti* Maus III (accession number: ERR4618952) was used as an outgroup to obtain the rooted tree. The substitution model was calculated with ModelTest-NG v0.1.7 [37]. Phylogenetic reconstruction was performed with RAxML-NG [38] using the maximum likelihood method and a bootstrap cut-off = 0.01. Visualization of the phylogenetic tree was achieved with iTOL v6.6 [39]. The transmission groups were determined by evaluating a distance of 12 SNPs between strains due showing the highest agreement between epidemiological research and genomic data [40, 41] The transmission cluster sizes were classified according to the number of shared isolates as follows: small if it had fewer than three isolates, medium if it was composed of three to five isolates and large if it had more than five isolates.

### Predicting susceptibility and drug resistance

The web-based tools TB-Profiler v5.0 (https://github.com/jodyphelan/TBProfiler), PhyReSse v1.0 (The Phylo-Resistance-Search-Engine) (https://bioinf.fz-borstel.de/mchips/phyresse/), Mykrobe v0.12.2 (https://www.mykrobe.com/), KvarQ v0.12.2 (https://kvarq.readthedocs.io/en/latest/index.html) and SAM-TB (https://samtb.uni-medica.com/index) were used to predict canonical mutations in genes related to resistance, such as rifampicin (RIF), isoniazid (INH), pyrazinamide (PZA), and ethambutol (EMB) (first-line drug) and fluoroquinolone (ciprofloxacin, levofloxacin, moxifloxacin, oxifloxacin, FQ), streptomycin (STR), ethionamide (ETH), and aminoglycosides (amikacin, AMK, kanamycin, KM, capreomicin, CAP) (second-line drugs). All programs were run under the default parameters using the high-quality reads from the 88 samples.

### Statistical analysis

Global and drug-specific sensitivities, specificities, positive predictive values (PPV) and negative predictive values (NPV) were computed using MedCalc® Statistical Software version 22.013 (MedCalc Software Ltd, Ostend, Belgium; https://www.medcalc.org; 2023) with a 95% confidence interval. For global analysis, we considered the tools capacity for detecting any resistance-related mutations that permit inclusion of the isolate/patient in a surveillance and drug scheme adjustment. The diagnostic results based on DST were used as a reference for the analysis.

### Ethics statements

Our study was reviewed and approved by the ethics committee of University Espíritu Santo under code 2022-001A. The research was conducted in accordance with the ethical principles outlined in the Declaration of Helsinki. Informed consent of the patients was not required since our study worked with isolates from a collection. The ethics committee determined the exception of informed consent because the isolates were anonymized, and no data on the patients were disclosed. The present work is based on INSPI-LIP permission to use the positive samples.

## Results

The drug susceptibility of *M*. *tuberculosis* isolates obtained from patients between 2019 and 2021 was assessed via drug susceptibility testing (DST) for four first-line drugs (rifampicin, isoniazid, pyrazinamide, and ethambutol), three second-line drugs (streptomycin, levofloxacin, and moxifloxacin), and three injectable drugs (kanamycin, amikacin, and capreomycin). Of the 88 samples, 22 were identified as susceptible, while 66 were found to be drug resistant (DR-TB) and further categorized as MDR (n = 46), polyresistant (n = 13), rifampicin and fluoroquinolone resistant (n = 5), and rifampicin monoresistant (n = 2). Among the isolates, 26.1% were from female patients, with a median age of 39.2 ± 14.5 years (range 21–73 years), and 73.9% were from male patients, with a median age of 40.3 ± 15.0 years (range 11–74 years). With regard to treatment history, 37.5% of the isolates were from treatment-naive cases (45.5% were drug-resistant), 23.9% were from patients receiving treatment (90.5% were drug-resistant), and 38.6% were from previously treated cases (94.1% were drug-resistant). The majority of the drug-resistant isolates (59.1%) were obtained from patients in Guayaquil, one of the most important economic centers in Ecuador, followed by Babahoyo, El Empalme, and Quito (4.5%, 3.4%, and 2.3%, respectively). A small number of cases were detected in Chone, Duran, Guaranda, Machala, and Nueva Loja (1.1% each) (S1 Table).

**Fig 1:**
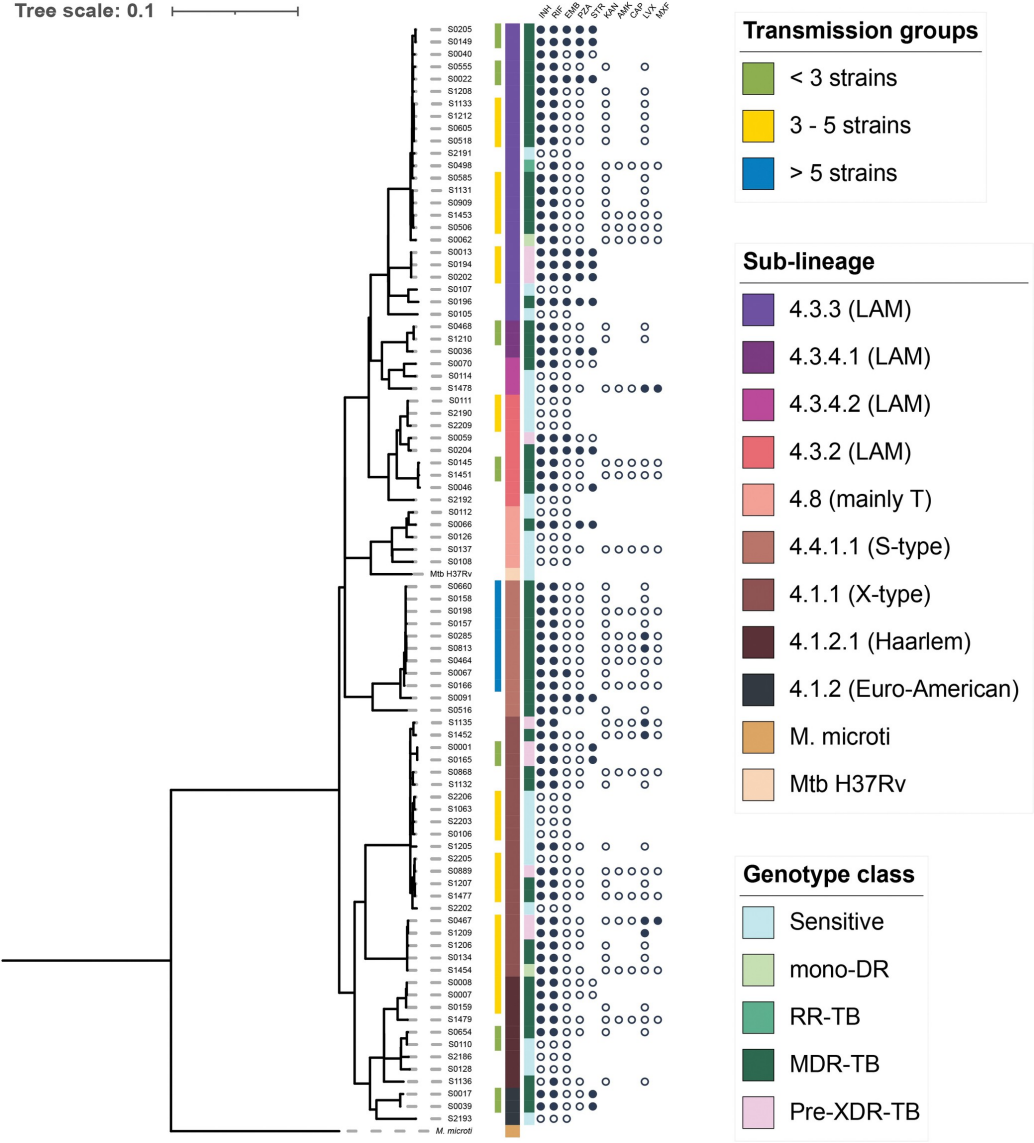
Phylogenetic reconstruction of the 88 Mtb isolates from Ecuador. A total of 5,586 SNPs were used to reconstruct the phylogenetic tree using the maximum likelihood method, the GTR substitution model and a bootstrap cut-off of 0.01. Metadata include i) transmission groups (lines green, yellow, and blue), ii) sublineage classification, iii) global genotypic class of drug resistance and iv) DST results of first- and second-line antibiotics (full circle = resistant; empty circle = sensitive; missing circle = not tested).

The MTBseq pipeline was used to assess the phylogenetic relationships among the 88 isolates by analysing 5586 SNPs. The results indicated a high diversity of circulating isolates in Ecuador, with LAM sublineages being the most predominant (44.3%), followed by X-type (23.9%), S-type and Haarlem (11.4%, each), as shown in Fig 1.

Although no association was found between sublineages and drug sensitivity, it is worth noting that out of the 10 Haarlem strains, 7 (70.0%) displayed MDR resistance, while three were sensitive. Cluster analysis identified 54 isolates (61.4%) grouped into 16 potentially molecular-related transmission clusters (with at least two isolates and ≤12 SNP differences), while 34 isolates were singletons (S1 Table, Fig 1). Seven clusters were considered small (< 3 isolates), eight medium (3–5 isolates), with the most prevalent being clusters 13 and 16 (five isolates each), and one large cluster with nine isolates (group 01). Pre-XDR isolates were distributed in four clusters, with three isolates in cluster 10 and two isolates each in clusters 11 and 16. The remaining pre-XDR isolates did not form clusters, and one isolate was unique. Notably, the only large cluster consisting of nine isolates was entirely composed of the S-type sublineage (S1 Table, Fig 1).

The performance and functionalities of five web-based tools (TB-Profiler v5.0, PhyResSE v1.0, Mykrobe v0.12.2, KvarQ v0.12.2, and SAM-TB) were evaluated by comparing the phenotypic resistance profile of the 88 strains (S2 Table). Descriptive statistics such as sensitivity, specificity, accuracy, percentage of agreement and kappa coefficient were calculated and are presented in Table 1.

**Table 1:** Comparative statistical analysis of five software programs for predicting anti-TB drug resistance.

Statistic	Mykrobe	KvarQ	TB-Profiler	PhyResSE	SAM-TB
**Sensitivity**	0.97 (0.89 to 1.00)	0.92 (0.83 to 0.97)	0.97 (0.89 to 1.00)	0.97 (0.89 to 1.00)	0.97 (0.89 to 1.00)
**Specificity**	1.00 (0.85 to 1.00)	1.00 (0.85 to 1.00)	1.00 (0.85 to 1.00)	1.00 (0.85 to 1.00)	1.00 (0.85 to 1.00)
**Positive Likelihood Ratio**					
**Negative Likelihood Ratio**	0.03 (0.01 to 0.12)	0.08 (0.03 to 0.18)	0.03 (0.01 to 0.12)	0.03 (0.01 to 0.12)	0.03 (0.01 to 0.12)
**Positive Predictive Value**	1.00 (0.94 to 1.00)	1.00 (0.94 to 1.00)	1.00 (0.94 to 1.00)	1.00 (0.94 to 1.00)	1.00 (0.94 to 1.00)
**Negative Predictive Value**	0.92 (0.74 to 0.98)	0.82 (0.65 to 0.91)	0.92 (0.74 to 0.98)	0.92 (0.74 to 0.98)	0.92 (0.74 to 0.98)
Accuracy (*)	0.98 (0.92 to 1.00)	0.94 (0.87 to 0.98)	0.98 (0.92 to 1.00)	0.98 (0.92 to 1.00)	0.98 (0.92 to 1.00)
**% of agreement**	0.98	0.94	0.98	0.98	0.98
**Cohen’s kappa**	0.941	0.859	0.941	0.941	0.941
	Almost perfect agreement	Substantial agreement	Almost perfect agreement	Almost perfect agreement	Almost perfect agreement

Numbers in brackets represent the corresponding 95% confidence intervals. Cohen’s kappa: 0.01–0.20: slight agreement; 0.21–0.40: fair agreement; 0.41–0.60: moderate agreement; 0.61–0.80: substantial agreement; 0.81–1.00: almost perfect or perfect agreement

All the evaluated programs exhibited good sensitivity and specificity globally, but their performance varied for preconditions of resistance to specific drugs. The highest level of agreement was observed for isoniazid on all programs (agreement and kappa coefficient above 93% and k = 0,840), while KvarQ had the lowest agreement for rifampicin (agreement 90.91% and k = 0,784).

In terms of detecting specific resistances, all tools showed good detection values for isoniazid and rifampicin. However, the detection of ethambutol and pyrazinamide varied across the different tools. TB-profiler and SAM-TB demonstrated adequate detection values, while KvarQ and PhyResSE exhibited lower quality values. In summary, TB-profiler and SAM-TB were found to be the most effective in detecting resistance to first-line antibiotics, whereas KvarQ was the least effective. For streptomycin, Mykrobe and KvarQ showed fair agreement in detecting resistance. However, all tools had difficulties detecting resistance to fluoroquinolones, particularly levofloxacin, and only KvarQ and SAM-TB presented the best agreement, while for moxifloxacin, only KvarQ showed moderate agreement. The statistical parameters for kanamycin, amikacin, and capreomycin could not be determined because only sensitive isolates were identified phenotypically (Table 2).

**Table 2:** Sensitivity, specificity and accuracy of ten drugs of anti-TB drug resistance analysed by WGS plus web-based tools compared with DST.

Mykrobe	Isoniazid (n = 87)	Rifampicin (n = 88)	Ethambutol (n = 87)	Pyrazinamide (n = 64)	Streptomycin (n = 21)	Kanamycin (n = 45)	Amikacin (n = 21)	Capreomycin (n = 21)	Levofloxacin (n = 46)	Moxifloxacin (n = 21)
Sensitivity	95.2 (86.7 to 99.0)	95.5 (87.3 to 99.1)	100.0 (71.5 to 100.0)	83.3 (51.6 to 97.9)	56.3 (29.9 to 82.3)				42.9 (9.9 to 81.6)	50.0 (1.3 to 98.7)
Specificity	96.0 (79.7 to 99.9)	100.0 (84.6 to 100)	86.8 (77.1 to 93.5)	94.2 (84.1 to 98.8)	100.0 (47.8 to 100)	95.6 (84.9 to 99.5)	95.2 (76.2 to 99.9)	95.2 (76.2 to 99.9)	97.4 (86.5 to 99.9)	89.5 (66.9 to 98.7)
Accuracy	95.5 (88.8 to 98.8)	96.6 (90.4 to 99.3)	88.5 (79.8 to 94.4)	92.2 (82.7 to 97.4)	66.7 (43.0 to 85.4)				89.1 (76.4 to 96.4)	85.7 (63.7 to 97.0)
Cohen’s kappa	0.891	0.913	0.625	0.751	0.380				0.489	0.323
**TB-Profiler**										
Sensitivity	93.7 (84.5 to 98.2)	100.0 (94.2 to 100)	100.0 (71.5 to 100)	83.3 (51.6 to 97.9)	68.8 (41.3 to 89.0)				42.9 (9.9 to 81.6)	50.0 (1.3 to 98.7)
Specificity	96.0 (79.7 to 99.9)	84.6 (65.1 to 95.6)	85.5 (72.5 to 90.6)	86.5 (74.2 to 94.4)	100.0 (47.8 to 100)	95.6 (84.9 to 99.5)	95.2 (76.2 to 99.9)	95.2 (76.2 to 99.9)	97.4 (86.5 to 99.9)	89.5 (66.9 to 98.7)
Accuracy	94.3 (87.2 to 98.1)	95.5 (88.8 to 98.8)	87.4 (75.8 to 91.8)	85.9 (75.0 to 93.4)	76.2 (52.8 to 91.8)				89.1 (76.4 to 96.4)	85.7 (63.7 to 97.0)
Cohen’s kappa	0.865	0.886	0.599	0.602	0.511				0.489	0.323
**PhyResSE**										
Sensitivity	95.2 (86.7 to 99.0)	97.0 (89.5 to 99.6)	90.9 (58.7 to 99.8)	75.0 (42.8 to 94.5)	68.8 (41.3 to 89.0)				42.9 (9.9 to 81.6)	50.0 (1.3 to 98.7)
Specificity	96.0 (79.7 to 99.9)	100.0 (84.6 to 100)	85.5 (75.6 to 92.6)	86.5 (74.2 to 94.4)	100.0 (47.8 to 100)	95.6 (84.9 to 99.5)	95.2 (76.2 to 99.9)	95.2 (76.2 to 99.9)	97.4 (86.5 to 99.9)	89.5 (66.9 to 98.7)
Accuracy	95.5 (88.8 to 98.8)	97.7 (92.0 to 99.7)	86.2 (77.2 to 92.7)	84.4 (73.1 to 92.2)	76.2 (52.8 to 91.8)				89.1 (76.4 to 96.4)	85.7 (63.7 to 97.0)
Cohen’s kappa	0.891	0.941	0.523	0.545	0.511				0.489	0.323
**KvarQ**										
Sensitivity	92.1 (82.4 to 97.4)	87.9 (77.5 to 94.6)	72.7 (39.0 to 94.0)	66.7 (34.9 to 90.1)	56.3 (29.9 to 82.3)				42.9 (9.9 to 81.6)	50.0 (1.3 to 98.7)
Specificity	96.0 (79.7 to 99.9)	100.0 (84.6 to 100)	93.4 (85.3 to 97.8)	88.5 (76.6 to 95.7)	100.0 (47.8 to 100)	100.0 (92.1 to 100)	100.0 (83.9 to 100)	100.0 (83.9 to 100)	100.0 (91.0 to 100)	94.7 (74.0 to 99.9)
Accuracy	93.2 (85.8 to 97.5)	90.9 (82.9 to 96.0)	90.8 (82.7 to 96.0)	84.4 (73.1 to 92.2)	66.7 (43.0 to 85.4)				91.3 (79.2 to 97.6)	90.5 (69.6 to 98.8)
Cohen’s kappa	0.840	0.784	0.614	0.518	0.380				0.56	0.447
**SAM-TB**										
Sensitivity	95.2 (86.7 to 99.0)	97.0 (89.5 to 99.6)	90.9 (58.7 to 99.8)	100.0 (73.5 to 100)	75.0 (47.6 to 92.7)				71.4 (29.0 to 96.3)	50.0 (1.3 to 98.7)
Specificity	96.0 (79.7 to 99.9)	100.0 (84.6 to 100)	85.5 (75.6 to 92.6)	84.6 (71.9 to 93.1)	100.0 (47.8 to 100)	100.0 (92.1 to 100)	95.2 (76.2 to 99.9)	95.2 (76.2 to 99.9)	97.4 (86.5 to 99.9)	79.0 (54.4 to 94.0)
Accuracy	95.5 (88.8 to 98.8)	97.7 (92.0 to 99.7)	86.2 (77.2 to 92.7)	87.5 (76.9 to 94.5)	81.0 (58.1 to 94.6)				93.5 (82.1 to 98.6)	76.2 (52.8 to 91.8)
Cohen’s kappa	0.891	0.941	0.523	0.674	0.588				0.732	0.173

Cohen’s kappa: 0.01–0.20: slight agreement; 0.21–0.40: fair agreement; 0.41–0.60: moderate agreement; 0.61–0.80: substantial agreement; 0.81–1.00: almost perfect or perfect agreement.

By genomic analysis of 88 phenotypic isolates, we identified genotypically 59.1% of strains MDR (52/88), 11.36% pre-XDR, 1.13% Monoresistant (1/88 each for HR and RR), and 27.3% Sensitive (24/88). Diversity in the genotypic resistance profile from the isolates was observed among the drugs assessed. Mykrobe, PhyResSE and SAM-TB detected the highest proportion of resistance to isoniazid, with 61 out of 88 isolates (69.3%). Similarly, PhyResSE and SAM-TB detected 64 out 88 isolates (72.7%) for rifampicin, while for ethambutol with 22 out 87 isolates (25.3%) and pyrazinamide with 20 out 64 isolates (31.3%), TB-profiler and SAM-TB were the best; however, the lowest resistance proportions were mainly described by KvarQ.

It is noteworthy that all programs detected resistance to second-line drugs that were not detected by DST. This may be associated with the slower growth of some of the isolates at the limit of the DST detection range. In particular, all programs detected a range of 2 to 5 isolates with mutations associated with resistance to kanamycin, amikacin and capreomycin, none of which were previously detected by DST. In terms of fluoroquinolones, between 10 and 12 isolates were characterized as resistant to moxifloxacin and levofloxacin by the programs, while DST detected only 2 and 7 resistant isolates, respectively. Overall, KvarQ detected less resistance, while SAM-TB was the program that detected more resistance, followed by PhyResSE and TB-Profiler (Table 3).

**Table 3:** Comparison of the number of cases of resistance detection by phenotypic (DST) and genotypic web-based tools for ten TB anti-drugs.

Treatment drugs	DST n (%)	Mykrobe n (%)	TB-profiler n (%)	PhyReSse n (%)	KvarQ n (%)	SAM-TB n (%)
**Isoniazid**	63 (71.6)	61 (69.3)	60 (68.2)	61 (69.3)	59 (67.1)	61 (69.3)
**Rifampicin**	66 (75.0)	63 (71.6)	62 (70.5)	64 (72.7)	58 (65.9)	64 (72.7)
**Ethambutol**	11 (12.5)	21 (23.9)	22 (25.0)	21 (23.9)	13 (14.7)	21 (23.9)
**Pyrazinamide**	12 (13.6)	14 (15.9)	18 (20.5)	18 (20.5)	15 (17.0)	21 (23.9)
**Streptomycin**	16 (18.2)	21 (23.9)	23 (26.1)	23 (26.1)	19 (21.6)	24 (27.3)
**Kanamycin**	0 (0.0)	5 (5.7)	5 (5.7)	5 (5.7)	2 (2.3)	5 (5.7)
**Amikacin**	0 (0.0)	3 (3.4)	3 (3.4)	3 (3.4)	2 (2.3)	2 (2.3)
**Capreomycin**	0 (0.0)	3 (3.4)	3 (3.4)	3 (3.4)	2 (2.3)	3 (3.4)
**Levofloxacin**	7 (8.0)	11 (12.5)	10 (11.4)	11 (12.5)	10 (11.4)	12 (13.6)
**Moxifloxacin**	2 (2.3)	11 (12.5)	10 (11.4)	11 (12.5)	10 (11.4)	12 (13.6)

### Polymorphisms associated with drug resistance identified by whole-genome sequencing and web-based tools

Whole-genome sequencing and web-based tool analysis of the 88 isolates revealed a total of 60 mutations distributed among 4 promoter regions, 5 intergenic regions, and 51 coding regions leading to changes in the reading frame. The majority of the mutations were single nucleotide changes, although 8 insertions and 4 deletions were also detected. Notably, three of the deletions were of considerable length, with one at 193 nt in the gid gene and two affecting the *pncA* and Rv2041c-Rv2042c genes at 891 and 833 nt, respectively. The *pncA* and *rpoB* genes associated with resistance to pyrazinamide and rifampicin, respectively, had the highest number of mutations, with *pncA* showing 13 mutations, two insertions of 4 nt each, and one deletion of 833 nt, while *rpoB* had 14 nonsynonymous mutations, some of which occurred in the same codon. Position 445 of the *rpoB* gene was the most variable codon, with five different configurations, followed by position 406 of the *embB* gene with three different mutations. Additionally, the *embB*, *gyrA*, and *rrs* genes, which confer resistance to ethambutol, fluoroquinolones, and streptomycin, respectively, had at least 4 mutations (Table 4, S3 Table).

**Table 4:** Mutational changes in resistant isolates of *Mycobacterium tuberculosis* identified by WGS.

RIF		INH		EMB		PZA		STR		KAM		FQ		ETH	
***rpoA***	T187A	***katG***	S315T	***embA***	-16C>T	***pncA***	-10A>G	***rpsL***	K43R	***eis***	-12C>T	***gyrA***	D89G	***ethA***	1222delT
***rpoB***	P45S		S315N	***embB***	M306I		V7A	***rrs***	514A>C	***rrs***	1401A>G		A90V	***ethR***	A95T
	V170F				M306V		D8H		517C>T				D94A		F110L
	K274N				D354A		H43P		888G>A				D94N	***inhA/*** ***fabG1*** *	-15C>T
	T400A						A46V		906A>G			***gyrB***	N499D		
	L430P				G406A		D49A	***gid***	102delG						
	D435V D435Y	***inhA/*** ***fabG1*** *	-15C>T		G406D		H51R								
	H445C				G406S		P69L		148_340del193						
	H445P						H82R								
	H445Q						S104G								
	H445R						G108E								
	H445Y						V125F H137Y								
	K446Q						M175I								
	S450L I491V						254insGGGC								
***rpoC***	W484G						292insCCCG								
	I491V						503-*833del								
						***pncA*, Rv2041c-Rv2042c**	2287848_2288738del891								

*inhA promoter mutations include mutations in the *fabG1* open reading frame (ORF) because they create alternative promoters for *inhA* and mutations upstream of *fabG1* because they act as promoters of the entire operon, which includes *inhA*. Abbreviations: rifampicin (RIF), isoniazid (INH), pyrazinamide (PZA), ethambutol (EMB) (first-line drug) and fluoroquinolone (ciprofloxacin, levofloxacin, moxifloxacin, oxifloxacin, FQ), streptomycin (STR), ethionamide (ETH), and aminoglycosides (amikacin, AMK, kanamycin, KM, capreomicin, CAP) (second-line drugs).

### Polymorphisms conferring rifampicin resistance

Mutations in the *rpoB*, *rpoC*, and *rpoA* genes were frequently identified in the 66 isolates defined as phenotypically resistant to rifampicin through whole-genome sequencing. The S450L *rpoB* mutation was the most prevalent (44/64), followed by D435V (6/64), V170F (3/64), D435Y, H445C/R/Y, and L430P (1/64 each). One isolate showing the V170F and L430P mutations and three isolates with the L430P and H445Q mutations were also identified. TB-profiler, PhyResSE and SAM-TB were able to identify mutations in one isolate (S0555_Mtb_Ec) with H445P and K446Q, while SAM-TB was able to identify in one isolate (S0506_Mtb_Ec) the mutations S450L plus K274N and S450L plus P45S (S0196_Mtb_Ec), while the others only detected S450L. KvarQ was able to identify isolates with the compensatory mutations W484G (S0001_Mtb_Ec and S0165_Mtb_Ec) and I491V (S1131_Mtb_Ec) in *rpoC* and the T187A (S0091_Mtb_Ec) mutation in the *rpoA* gene, while PhyResSE detected the compensatory mutation I491V (S0204_Mtb_Ec) in the *rpoB* gene. None of the sensitive isolates had any characterized mutations.

### Polymorphisms conferring isoniazid and ethionamide resistance

Out of 63 clinical isolates where isoniazid resistance was reported by phenotypic susceptibility testing, at least one known isoniazid-resistance mutation was found in 61. For isolates S1135_Mtb_Ec and S1205_Mtb_Ec with DST-detected resistance, the 426C>T mutation was found, but it was not previously reported for isoniazid. One isolate reported as sensitive by DST, S1136_Mtb_Ec, had one resistance-related mutation. The most prevalent mutation related to isoniazid resistance was S315T in the *katG* gene (55/63), particularly S315N, which was detected in only one isolate (S0070_Mtb_Ec). Five isolates presented the mutation -15C>T in the *fabG1*/*inhA* promoter gene related to isoniazid/ethionamide resistance. Other ethionamide resistance mutations were reported by TB-profiler and SAM-TB in the *ethR* gene (A95T and F110L mutations) and the *ethA* gene (the 1222delT deletion). For three isolates with DST-detected isoniazid resistance, no known mutations related to this resistance were found.

### Polymorphisms conferring ethambutol resistance

The analysis of the studied isolates revealed the presence of multiple mutations in the *embB* and *embA* genes, including M306I/V, D354A, G406A/D/S, and a -16C>T substitution. M306I was the most frequently observed mutation, found in 11 out of 22 isolates, often in combination with G406D and D354A. Additionally, M306V and G406S were each observed in four isolates. It is noteworthy that mutations in the *embB* and *embA* genes were identified in 11 initially ethambutol-sensitive isolates as determined by DST.

### Polymorphisms conferring pyrazinamide resistance

A diverse range of mutations in the *pncA* gene associated with pyrazinamide resistance was observed, including 8 insertions, 1 deletion, and 14 single nucleotide polymorphisms that spanned the entire *pncA* gene, including its promoter region. The most frequently occurring mutations were H82R and V125F (3/21), followed by H51R and G108E (2/21), along with some unique mutations, including V7A, D8H, H43P, A46V, D49A, P69L, S104G, H137Y, and M175I, and a substitution of -10A>G in the promoter region. Two isolates had larger deletions, one spanning 891 nucleotides encompassing the *pncA* and Rv2041c-Rv2042c genes and the other having an 833-nucleotide deletion within the *pncA* gene (503-*del833). Furthermore, specific insertions were identified in two isolates: 292GC, 293CC, 294CC, and 295GG in S0036_Mtb_Ec and 254TG, 255GG, 256GG, and 257CC in S0040_Mtb_Ec. Remarkably, mutations in the *pncA* gene were identified in 8 isolates initially characterized as pyrazinamide-sensitive by DST. Additionally, mutations in the rpsA gene were detected in 2 isolates, but these were not associated with pyrazinamide resistance. In isolate S2193_Mtb_Ec, only PhyResSE detected the H137Y mutation in pncA, however this is not thought to be associated with resistance.

### Polymorphisms conferring fluoroquinolone resistance

Thirteen isolates were found to have mutations associated with resistance to FQ. The most common mutation, present in six out 13 isolates, was A90V, followed by D94N (three isolates) and D89G (one isolate), all located in the *gyrA* gene. One DST-sensitive isolate had a combination of two mutations, D94A in the *gyrA* gene and N499D in *gyrB*. However, one isolate that was identified as resistant to levofloxacin and moxifloxacin by DST did not have any resistance-related mutations. Two isolates resistant to levofloxacin but sensitive to moxifloxacin were detected to have resistance-related mutations only by SAM-TB. No mutations related to FQ resistance were found in the majority of the isolates identified as sensitive by DST.

### Polymorphisms conferring aminoglycoside resistance

Among the 28 isolates identified as resistant to streptomycin, the K43R mutation in the *rpsL* gene was the most common, found in 16 isolates, while the K88R mutation was the least frequent, present in only one isolate. Mutations in the *rrs* gene were also observed, including 514A>C (3/28), 517C>T, 888G>A, and 906A>G. In addition, deletions in the gid gene were identified, including 102delG and 148_340del193. Interestingly, even isolates classified as sensitive to kanamycin, amikacin, and capreomycin by DST had the known resistance-associated substitution 1401A>G in the *rrs* gene in three isolates and two isolates exhibiting the -12C>T mutation in the *eis* gene, which is associated with kanamycin resistance.

## Discussion

Although sequencing-based diagnostic information for *M*. *tuberculosis* has been available for several years and recent methodological advancements have enabled the use of direct whole-genome sequencing for accurate prediction of drug resistance, this is the first study to assess and compare the effectiveness of whole-genome sequencing and user-friendly web-based tools in inferring drug resistance in tuberculosis drug susceptibility surveillance in Ecuador. The study utilized isolates obtained from nine cities, with Guayaquil being the most representative city, accounting for 59.1% of the isolates. This high number of tuberculosis cases detected in Guayaquil may be attributed to the high mobility of people from other provinces for trade or work, in addition to the presence of leading health centers for monitoring this pathogen.

Accelerating diagnosis and administering appropriate treatment based on the rapid identification of resistant strains and transmission clusters are crucial in reducing the incidence of tuberculosis [4]. In our study, whole-genome sequencing detected 97% of phenotypically resistant isolates accurately and identified mutations associated with resistance in 18.2% of phenotypically sensitive isolates within a week. However, discordant results (2.3%) were observed, likely due to factors such as critical concentration in some DST systems, mutations outside the drug resistance regions, silent mutations, and heteroresistance, which are missed by conventional methods [42–44].

Whole-genome sequencing (WGS) has been evaluated as a useful tool to quantify transmission clusters, with a threshold of 12 SNPs. In our study, using 12 SNPs, we identified sixteen mostly medium-sized transmission clusters (3–5 isolates), indicating local aggregations, and showed a high transmission rate (54/88, 61.4%). This is in contrast to previous studies where a low transmission rate was reported using MIRU-VNTR [45, 46] or using WGS in other localities [47, 48]. This could be due to a large percentage of cluster of two isolates (43.8%) in our results or the highest SNPs umbral used in other studies (15 SNPs).

WGS is a powerful tool that can simultaneously differentiate *M*. *tuberculosis* species, detect drug resistance, identify genetic diversity, and track transmission dynamics [49–51]. However, its use in clinical practice is still limited due to challenges in analysing genomic sequencing data, including the lack of standardized criteria for identifying noncanonical mutations and selecting diagnostic criteria for resistance [22]. The WHO has updated its TB treatment guidelines, including related mutations for drug-susceptible, single drug-resistant, MDR-TB, and XDR-TB, to improve web-based tools [23]. Despite several TB-specific genome browsers and WGS analysis tools being available, their use in surveillance programs is limited in countries such as Ecuador due to inadequate access to computational infrastructure, funding, and technical expertise. Clinicians and public health officials may also lack specialized knowledge to interpret genomic data, further impeding their adoption. Thus, promoting the use of these tools and improving their accessibility and usability for routine clinical and public health purposes is essential.

To evaluate their usefulness, we compared the accuracy, sensitivity, and specificity of five tools for inferring the resistance of *M*. *tuberculosis* isolates against phenotypic tests. Our results showed that globally, TB-profiler, PhyResSE, Mykrobe, and SAM-TB have a better ability to identify any resistance pattern in *M*. *tuberculosis* isolates, with an overall sensitivity, specificity, and accuracy of 97.0%, 100.0% and 97.7%, respectively. Notably, SAM-TB, the most recently developed tool, combines features of other pipelines and improves the detection and interpretation of resistance, similar to a panel that shows variations in isolates analysed, including statistical data, quality control, drug resistance, phylogenetic tree reconstruction and pairwise SNP distance into isolates and references. Although these web-based tools demonstrated good overall performance in detecting drug resistance in *Mycobacterium tuberculosis* isolates with conventional DST, they also revealed limitations in the predictive power of some mutations in their catalogue. In addition, the tools showed varying levels of sensitivity and specificity due to differences in the sets of mutations used and their underlying genotyping pipelines. Notably, this would indicate that the predictive power of these tools should be considered with varying degrees of confidence depending on the predictive performance of the detected markers [15, 52]; whence, finding a solution to maintain and standardized update the automated pipelines to WHO guidelines is crucial to ensure their continued effectiveness.

Drug resistance in *M*. *tuberculosis* is primarily mediated by mutations in specific gene targets. Therefore, identifying the responsible or strongly associated SNPs is crucial for effective diagnosis and treatment [8, 25, 53, 54]. In our study, we identified 17 resistance-associated genes out of 42 candidate genes, which comprised a total of 69 mutations. Canonical mutations associated with resistance have been previously identified in various studies, along with deletions that can affect gene functionality or promote bacterial transmission [51, 55–63].

In first-line drugs, numerous studies have demonstrated that the rifampicin resistance-determining region (RRDR) in the *rpoB* gene accurately predicts rifampicin resistance [56, 64, 65]. In our study, we discovered that the S450L mutation was the most significant in predicting resistance and linked to high levels of resistance [66, 67]. Additionally, compensatory mutations in the *rpoC* gene (W848G and I491V) and the *rpoA* gene (T187A) were associated with multidrug-resistant (MDR) transmission [3, 68–71]. These mutations may contribute to the faster emergence of MDR strains and highlight the importance of monitoring these genetic changes to prevent the spread of drug resistance. Likewise, mutations in various genes have been associated with conferring resistance to isoniazid [55, 72, 73] and ethionamide [59, 74, 75]. Our study found a higher prevalence of mutations in the *katG* gene (88.8%), which is consistent with prior research [55, 73, 76]. On the other hand, mutations in the *embCAB* gene operon are frequently involved in ethambutol resistance [57, 77–80]. We found that a higher number of mutations occurred in the *embB* gene, which is consistent with previous studies. Additionally, all the mutations we identified as related to pyrazinamide resistance coincided with studies pointing to the importance of the promoter and the *pncA* gene [4, 57, 62, 81, 82]. Our study confirms the importance of monitoring mutations in the RRDR of the *rpoB* gene and the prevalence of mutations in the *katG*, *embB* and *pncA* genes for predicting isoniazid, ethambutol and pyrazinamide resistance, respectively. We also highlight the significance of compensatory mutations and the need to monitor them to prevent the emergence and spread of MDR strains.

For second-line drugs, our analysis showed that the majority (92.3%) of mutations associated with fluoroquinolone resistance were located specifically in the quinolone resistance-determining region (QRDR), consistent with previous reports linking mutations in these regions to cross-resistance to all FQs [74, 83–85]. Interestingly, one resistant isolate did not show any mutations in the *gyrA* or *gyrB* genes, suggesting the involvement of other mechanisms, such as efflux pumps, in generating fluoroquinolone resistance [86]. Similarly, 60.7% of mutations observed in the *rpsL* gene were the k43R mutation, which has been reported as the most recurrent streptomycin resistance-associated mutation [51, 85, 87]. In our study, we analysed the *rrs* gene and identified several substitutions associated with streptomycin (positions 514, 517, 888, and 906) or linked to cross-resistance to kanamycin, capreomycin, or amikacin (1401A>G). It is worth noting that this mutation has been found to confer high levels of resistance in previous studies [8, 72, 88–92]. Moreover, our study identified two isolates with the -12C>T mutation in the *eis* promoter that have been associated with low-level kanamycin resistance, supporting previous research findings [93, 94]. Therefore, our findings support the importance of monitoring this mutation and other known substitutions in the *rrs* gene to detect and prevent the spread of second-line drug-resistant TB strains.

Our study also identified genetic alterations that may impact drug resistance. Among these alterations were large deletions in the *pncA* and gid genes, which could have implications for drug susceptibility. Furthermore, we detected nonresistance-conferring mutations in the *katG* (R463L, L427P), *kasA* (G269S), *ahpC* (51G>A), *pncA* (H137Y) and *gyrA* (E21Q, S95T, G668D) genes. While these mutations do not confer drug resistance, they may still be useful for characterizing the evolutionary history of *M*. *tuberculosis* [8, 95].

Our study faced limitations due to the COVID-19 pandemic, limited access to samples and epidemiological information, lack of access to phenotypic tests for all drugs and a small sample size. Due to the absence of complete susceptibility results for all drugs tested by DST, our study could lead to underestimation of the true magnitude of the resistance, especially for second-line drugs, as well as determine the real performance of tools used for detecting second-line drug resistance and loss of ability to determine rare resistance variants with low frequencies associated with intrinsic and extrinsic resistance. Future studies with larger sample sizes and broader coverage should be conducted to identify mutations in tuberculosis patients in other regions of Ecuador. The study suggests to using TB-specific genome browsers and WGS analysis tools such as SAM-TB to aid in genotyping, drug resistance, and transmission cluster identification. These tools should be implemented more routinely in TB surveillance programs, particularly in local conditions. The study highlights the potential of WGS technology and bioinformatic tools to improve TB diagnosis and treatment and ultimately reduce the global burden of TB.

## Supporting information

S1 Table Sociodemographic, clinical, and genotypic results in 88 isolates of *Mycobacterium tuberculosis*. (XLSX)

S2 Table Individual phenotypic and genotypic resistance pattern in 88 isolate of *Mycobacterium tuberculosis*. S: Sensitive, R: Resistance, ND: Not determined.
(XLSX)

S3 Table Mutation profile from 88 isolates of *Mycobacterium tuberculosis* by five web-based tools. (XLSX)
